# Feed microbiome: confounding factor affecting fish gut microbiome studies

**DOI:** 10.1038/s43705-022-00096-6

**Published:** 2022-02-02

**Authors:** Christian Karlsen, Dimitrios Tzimorotas, Espen Mikal Robertsen, Katrine Hånes Kirste, André Sture Bogevik, Ida Rud

**Affiliations:** 1 Nofima, Osloveien 1, 1433 Ås, Norway; 2 Centre for Bioinformatics, Faculty of Science and Technology, UiT The Arctic University of Norway, PO Box 6050 Langnes, N-9037 Tromsø, Norway

**Keywords:** Microbiome, Marine microbiology

## Abstract

There is an increasing interest in the impact of feed on the fish gut microbiome. Most of the studies are based on sequencing the bacterial housekeeping gene 16S rRNA from extracted total DNA, including resident and non-resident live bacteria as well as dead bacteria. It has not been a common practice to include the feed as control, although it contains various nutritious ingredients that microorganisms can use before or after feed preparation. Thus, study designs using digesta as a proxy for the intestinal microbiome raise the concern that composition of the gut microbiome might be biased by carry-over of microbial DNA from the feed itself. Here we report analysis of 15 feeds and representative intestinal digesta of Atlantic salmon *(Salmo salar*) from five independent case studies. This allowed us to identify “feed microbiomes” that were microbially diverse and shared taxa with digesta microbiomes. Digesta-specific microbiomes were identified, though they were mainly enriched by a few taxa, such as *Mycoplasma* and *Ruminococcaceae*. Overall, findings are consistent with a model wherein gut microbial profiles are to a different degree influenced by bacterial DNA present in the feed itself through a “feed microbiome” carry-over effect.

The fish intestinal tract is colonized by aerobic, facultative or obligate anaerobic bacteria, with Proteobacteria being the prominent phyla [1]. A comprehensive review on the role of the gut microbiome in cultured fish is described in Perry et al. [2]. Bacteria may be considered resident members of the gut, associated with the intestinal mucosa, autochthonous colonizers of the host gut, or transient organisms that are not closely associated with the surrounding digesta. Variability in microbial taxa between studies and individuals could relate to several factors such as feeding rates, condition factor, maturation, or environmental conditions. One factor that is often not evaluated in typical diet studies, is the feed itself. Feed can contain genetic material from the animal and plant species that were used in its preparation, as well as the microbiomes of those ingredients. The presence of DNA in animal feeds is thus expected. Typical commercial feed for Atlantic salmon may thus contain exotic microorganisms introduced with the ingredients, or from communities that developed during feed processing or storage. Although microbial contaminants may be killed before the final product is finished, their DNA or fragments of their DNA may remain. The present study addresses this concern.

Microbiome analysis of in total 15 feeds (F1–F15) from five independent case studies (Study 1–5) and representative digesta of individual fish consuming 12 of those feeds were analyzed using the 16S rRNA (V4) amplicon sequencing protocol from the Earth Microbiome Project. Sequence data were processed in QIIME2 as described in Supplementary Information. Overview of the five case studies and microbiome samples, including biological replicates, is presented in Supplementary Table S1 and S2, respectively. We identified feed microbiomes in all the feeds analyzed (Fig. 1), and with an alpha diversity of 140 ± 38 amplicon sequencing variants (ASVs) comparable to the distal intestinal digesta (DID) of 125 ± 93 ASVs (Supplementary Fig. S1). The taxonomic compositions of the different feed microbiomes were specific between the five case studies, but also feed specific, though to a lesser extent (Fig. 1A). Dominant genera found in feeds were *Photobacterium*, *Lactobacillus*, *Streptococcus*, *Weissella,* and *Paracoccus*, but their relative abundance varied among the five case studies and 15 feed types (Fig. 1B).

**Fig. 1:**
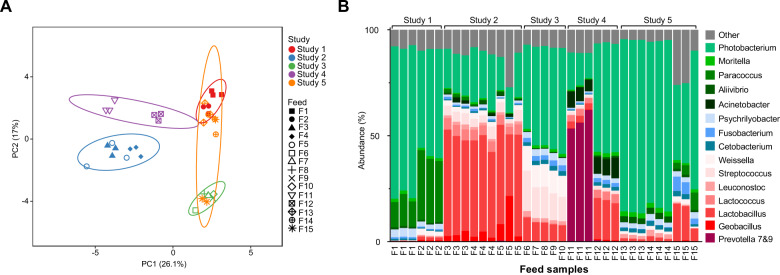
Feed microbiome comparison between the different case studies (*n* = 5) and feed types (*n* = 15). **A** PCA plot (scaled) and **B** taxonomic barplot. The representative studies (Study 1–5) and feed (F1–F15) numbers are indicated and includes biological replicates. Prediction ellipses represent the 95% confidence ellipse of the different sample sets. The barplot contains the 15 dominating genera (average abundance >1%). Other reflects the sum of all none dominating genera.

Comparison between all the different feed and DID microbiomes shows both shared (PC1) and different microbiome compositions (PC2) (Fig. 2A). Significant correlation was evident between 8 of the 12 feeds and their respective DID microbiomes (Supplementary Fig. S2, highlighted by red boxes). Even higher correlation was observed between the composition of the feed microbiome and microbiomes in digesta samples from the proximal and mid intestinal samples, though data are only available from study 1. Taxonomic comparison showed that several of the core genera in feed were frequently detected in the DIDs (Fig. 2B). This was especially the case for *Photobacterium*, detected with a frequency of 100% in the feeds vs 92% per DID category. Other core genera from the feed microbiome were also frequently detected in the DIDs, but only *Lactobacillus*, *Weissella*, and *Streptococcus* were among the dominants (mean DIDs >1%). *Photobacterium* was the major dominating taxa in both feed (46%) and digesta microbiomes (27%). The genus is common in fish microbiomes [1] and includes species that are both pathogenic and commensal to fish [3]. Only one *Photobacterium* ASV dominated across all the case studies (Supplementary Fig. S3), and the ASV data could not be used to determine if all the *Photobacterium* in the DIDs could be of feed origin. We confirmed the overlap between feed and DID microbiomes by a metagenomic analysis of one feed cohort (F5) of study 2 (Supplementary Fig. S4). The analysis also showed the existence of a more complex community pattern in the feed and DIDs, including detection of fungal taxa.

**Fig. 2:**
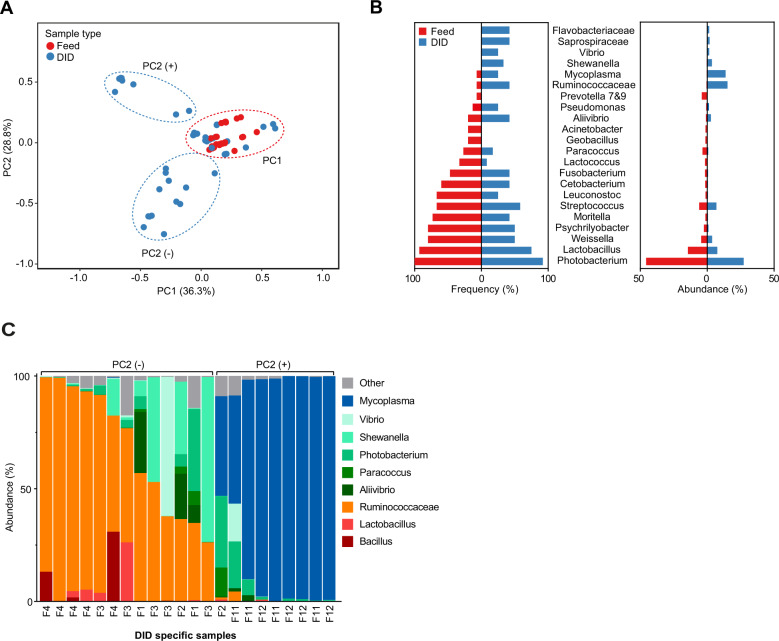
Feed and DID microbiome comparison. **A** PCA plot (unscaled) of all feed and DIDs. **B** Relative frequency and abundance of genera per feed (*n* = 15) and per representative DID (*n* = 12). **C** Taxonomic barplot of DID specific genera, differentiated into two groups (PC2- and PC2+), and colored coded according to phylum: Firmicutes (red/orange), Proteobacteria (green), Tenericutes (blue). Only dominating taxa were included in **B**, **C** (average abundance >1% across the feed or DID samples).

DID microbiomes that were separated from the feed microbiomes along PC2 in Fig. 2A, were enriched by only a few taxa within Firmicutes and Proteobacteria (PC2-) or Tenericutes (PC2+) (Fig. 2C). High dominance of *Ruminococcaceae* or *Mycoplasma* was observed within several of the DIDs, while other DIDs were dominated by one or several genera of *Aliivibrio, Shewanella*, *Photobacterium,* or *Vibrio*. The shifting dominance of these taxa corroborate other studies of the Atlantic salmon gut microbiome [4–6] where some individuals may become dominated by a few bacterial taxa [5, 7–9].

Here, we speculate that reported dietary effects may in part be biased by taxa from the feed microbiome as many aquaculture studies use digesta as a proxy for the intestinal microbiome. Dominant genera found in the 15 feeds of this study were *Photobacterium*, *Lactobacillus*, *Streptococcus*, *Weissella*, and *Paracoccus*. These remained as dominant taxa in many of the DID microbiomes and commensurate predominant taxa in digesta from other dietary studies of salmonids [10–13]. Indeed, a predominance of *Lactobacillus, Paracoccus, Streptococcus,* and *Weissella* has been identified from salmonid feeds [14–16]. It has also been suggested that several microbes, such as Lactobacillales, may not be active constituents in the fish gut [17]. Detection of predominant feed-associated taxa in many of the DID microbiomes is possibly due to a carry-over effect of bacterial DNA from the feed, exemplified with *Lactobacillus* (Supplementary Fig. S5). However, we assume that this will not influence the mucosa-associated microbiome to a similar extent as it is likely that the DNA will be transient and washed away during sample preparation of the mucosa. Indeed, we recognize that the digesta microbiome is different to the mucosa-associated microbiome [10, 17, 18], which is anticipated to affect hosts to a greater extent [19]. Thus, changes in digesta microbiomes attributed to diet could originate from other variations, i.e. influenced by different feeding rates, intestinal passage times, digestion rates [20–22], or fasting [23]. The overall implication of our study is that any intervention that alters feed consumption or changes digestion may have a direct effect on microbiome profile derived from the analysis, affecting our interpretation and thus conclusions about microbiome function.

Although much of what is known about fish gut microbiomes might come from true biological signals, many studies that have identified a distinct gut microbial community have not assessed carry-over effects of bacterial DNA from the feed in their findings. While the multifaceted nature of this situation limits our ability to clearly delineate the associations between feed and digesta microbiomes, we were able to describe novel associations between a “feed microbiome” and the DID across multiple cases.

## Supplementary information

Supplementary Material

## Data Availability

The raw sequence libraries can be accessed under the BioProject accession number PRJNA791377.

## References

[1] Egerton S, Culloty S, Whooley J, Stanton C, Ross RP (2018). The gut microbiota of marine fish. Front Microbiol.

[2] Perry WB, Lindsay E, Payne CJ, Brodie C, Kazlauskaite R (2020). The role of the gut microbiome in sustainable teleost aquaculture. Proc Biol Sci.

[3] Labella AM, Arahal DR, Castro D, Lemos ML, Borrego JJ (2017). Revisiting the genus Photobacterium: taxonomy, ecology and pathogenesis. Int Microbiol.

[4] Llewellyn MS, McGinnity P, Dionne M, Letourneau J, Thonier F, Carvalho GR (2016). The biogeography of the atlantic salmon (*Salmo salar*) gut microbiome. ISME J..

[5] Zarkasi KZ, Taylor RS, Abell GCJ, Tamplin ML, Glencross BD, Bowman JP (2016). Atlantic salmon (*Salmo salar* L.) gastrointestinal microbial community dynamics in relation to digesta properties and diet. Microbial Ecol..

[6] Dehler CE, Secombes CJ, Martin SAM (2017). Environmental and physiological factors shape the gut microbiota of Atlantic salmon parr (*Salmo salar* L.). Aquaculture.

[7] Holben WE, Williams P, Saarinen M, Särkilahti LK, Apajalahti JHA (2002). Phylogenetic analysis of intestinal microflora indicates a novel *Mycoplasma* phylotype in farmed and wild salmon. Microbial Ecol.

[8] Schmidt V, Amaral-Zettler L, Davidson J, Summerfelt S, Good C (2016). Influence of fishmeal-free diets on microbial communities in Atlantic salmon (*Salmo salar*) recirculation aquaculture systems. Appl Environ Microbiol.

[9] Lavoie C, Courcelle M, Redivo B, Derome N (2018). Structural and compositional mismatch between captive and wild Atlantic salmon (*Salmo salar*) parrs’ gut microbiota highlights the relevance of integrating molecular ecology for management and conservation methods. Evol Appl.

[10] Gajardo K, Jaramillo-Torres A, Kortner TM, Merrifield DL, Tinsley J, Bakke AM (2017). Alternative protein sources in the diet modulate microbiota and functionality in the distal intestine of Atlantic salmon (*Salmo salar*). Appl Environ Microbiol.

[11] Catalán N, Villasante A, Wacyk J, Ramírez C, Romero J (2018). Fermented soybean meal increases lactic acid bacteria in gut microbiota of Atlantic salmon (*Salmo salar*). *Probiotics and Antimicrobial*. Proteins.

[12] Rimoldi S, Terova G, Ascione C, Giannico R, Brambilla F (2018). Next-generation sequencing for gut microbiome characterization in rainbow trout (*Oncorhynchus mykiss*) fed animal by-product meals as an alternative to fishmeal protein sources. PLoS One.

[13] Wang J, Jaramillo-Torres A, Li Y, Kortner TM, Gajardo K, Brevik ØJ (2021). Microbiota in intestinal digesta of Atlantic salmon (Salmo salar), observed from late freshwater stage until one year in seawater, and effects of functional ingredients: a case study from a commercial sized research site in the Arctic region. Anim Microbiome.

[14] Huyben D, Roehe BK, Bekaert M, Ruyter B, Glencross B (2020). Dietary lipid:protein ratio and n-3 long-chain polyunsaturated fatty acids alters the gut microbiome of Atlantic salmon under hypoxic and normoxic conditions. Front Microbiol.

[15] Terova G, Gini E, Gasco L, Moroni F, Antonini M, Rimoldi S (2021). Effects of full replacement of dietary fishmeal with insect meal from *Tenebrio molitor* on rainbow trout gut and skin microbiota. J Anim Sci Biotechnol.

[16] Lorgen-Ritchie M, Clarkson M, Chalmers L, Taylor JF, Migaud H, Martin SAM (2021). A temporally dynamic gut microbiome in Atlantic salmon during freshwater recirculating aquaculture system (RAS) production and post-seawater transfer. Front Mar Sci..

[17] Legrand TPRA, Wos-Oxley ML, Wynne JW, Weyrich LS, Oxley APA (2021). Dead or alive: microbial viability treatment reveals both active and inactive bacterial constituents in the fish gut microbiota. J Appl Microbiol.

[18] Li Y, Bruni L, Jaramillo-Torres A, Gajardo K, Kortner TM, Krogdahl A (2021). Differential response of digesta- and mucosa-associated intestinal microbiota to dietary insect meal during the seawater phase of Atlantic salmon. Anim Microbiome.

[19] Glymenaki M, Singh G, Brass A, Warhurst G, McBain AJ, Else KJ (2017). Compositional changes in the gut mucus microbiota precede the onset of colitis-induced inflammation. Inflamm Bowel Dis.

[20] Parris DJ, Morgan MM, Stewart FJ (2019). Feeding rapidly alters microbiome composition and gene transcription in the clownfish gut. Appl Environ Microbiol.

[21] Zaldúa N, Naya DE (2014). Digestive flexibility during fasting in fish: a review. Comp Biochem Physiol Part A: Mol Integr Physiol.

[22] Gilannejad N, Silva T, Martínez-Rodríguez G, Yúfera M (2019). Effect of feeding time and frequency on gut transit and feed digestibility in two fish species with different feeding behaviours, gilthead seabream and Senegalese sole. Aquaculture.

[23] Xia JH, Lin G, Fu GH, Wan ZY, Lee M, Wang L (2014). The intestinal microbiome of fish under starvation. BMC Genom.

