# Supplementary material for: Feed microbiome: confounding factor affecting fish gut microbiome studies
Source: ISME Commun. 2022 Feb 2;2:14. doi: 10.1038/s43705-022-00096-6 (PMC9723547; doi:10.1038/s43705-022-00096-6)
Supplement: Supplementary file 1 — Supplementary Material [file 43705_2022_96_MOESM1_ESM.docx]

Supplementary Information

**Feed microbiome: Confounding factor affecting fish gut microbiome studies**

Christian Karlsen^a*^, Dimitrios Tzimorotas^a^, Espen Mikal Robertsen^b^, Katrine Hånes Kirste^a^, André Sture Bogevik^a^, Ida Rud^a^

^a^Nofima, Osloveien 1, 1433 Ås, Norway

^b^ Centre for Bioinformatics, Faculty of science and technology, UiT The Arctic University of Norway, PO Box 6050 Langnes, N-9037 Tromsø, Norway

^*^Corresponding author: christian.karlsen@nofima.no

**Materials and methods**

To better understand the effect of feed on the Atlantic salmon intestinal microbiome, fish and feeds from 5 different cases were used. Each case is named by study 1-5. Overview of the five case studies and microbiome samples, including biological replicates, is presented in Supplementary Table S1 and S2, respectively. Study 1, and 3 were trials performed at the Research Station for Sustainable Aquaculture Sunndalsøra, Norway. Study 2 was performed at VESO Vikan, Namsos, Norway. Study 4 used fish from two commercial farm sites located in the West of Norway. The two commercial systems are previously described [1], and fish obtain from both production systems at the last sample timepoint is utilized in this study. The fish were automatically fed to appetite by either commercial dry diets or feeds produced and formulated for experimental purposes. All husbandry practices at the commercial production facilities were conducted in accordance with national guidelines regarding animal welfare in addition to standard protocols for Lerøy Vest AS. Fish health and welfare status was regularly monitored by independent fish health veterinarians during production. Each experimental trial was approved by the Norwegian Animal Research Authority (NARA), and were conducted under regulations controlling experiments and procedures in live animals in Norway.

**Supplementary Table S1.** Overview of the five case studies and 15 feeds

|  | **Location** |  | **Seawater transfer** | **Rearing system** | **Diet duration** | **Sampling** | **Fish weight (g) ± SD** | **Condition factor** | **Temperature**  **(°C)**** |
| --- | --- | --- | --- | --- | --- | --- | --- | --- | --- |
| **Study and feed number** | **Latitude** | **Longitude** |  |  |  |  |  |  |  |
| Study 1 F1 | 62°40'02.8"N | 8°31'26.1"E | 04.10.2016 | Tanks FT water | 12 weeks | 29.01.2018 | 1739.7 ± 178.5 | 1.51 ± 0.09 | 12.5 → 9 |
| Study 1 F2 | 62°40'02.8"N | 8°31'26.1"E | 04.10.2016 | Tanks FT water | 12 weeks | 29.01.2018 | 1658.1 ± 165.2 | 1.43 ± 0.06 | 12.5 → 9 |
| Study 2 F3 | 64°32'49.5"N | 11°26'46.8"E | 22.08.2016 | Tanks FT water* | 11 weeks | 08.11.2016 | 433.5 ± 26.1 | 1.40 ± 0.06 | 12 |
| Study 2 F4 | 64°32'49.5"N | 11°26'46.8"E | 22.08.2016 | Tanks FT water* | 11 weeks | 08.11.2016 | 518.4 ± 95.4 | 1.50 ± 0.11 | 12 |
| Study 2 F5 | 64°32'49.5"N | 11°26'46.8"E | 22.08.2016 | Tanks FT water* | 11 weeks | 08.11.2016 | 520.5 ± 81.2 | 1.45 ± 0.12 | 12 |
| Study 3 F6 | 62°40'02.8"N | 8°31'26.1"E | 01.08.2017 | Tanks FT water | 10 weeks | 10.10.2017 | 317.3 ± 23.2 | 1.30 ± 0.04 | 13.0 ± 0.9 |
| Study 3 F7 | 62°40'02.8"N | 8°31'26.1"E | 01.08.2017 | Tanks FT water | 10 weeks | 10.10.2017 | 320.0 ± 62.7 | 1.26 ± 0.05 | 13.0 ± 0.9 |
| Study 3 F8 | 62°40'02.8"N | 8°31'26.1"E | 01.08.2017 | Tanks FT water | 10 weeks | 10.10.2017 | 304.7 ± 47.4 | 1.24 ± 0.07 | 13.0 ± 0.9 |
| Study 3 F9 | 62°40'02.8"N | 8°31'26.1"E | 01.08.2017 | Tanks FT water | 10 weeks | 10.10.2017 | 313.4 ± 54.8 | 1.31 ± 0.10 | 13.0 ± 0.9 |
| Study 3 F10 | 62°40'02.8"N | 8°31'26.1"E | 01.08.2017 | Tanks FT water | 10 weeks | 10.10.2017 | 283.6 ± 51.6 | 1.28 ± 0.08 | 13.0 ± 0.9 |
| Study 4 F11 | 60° 20′ 52.7′′N | 5° 38′ 29.3′′E | 28.04.2016 | Semi-closed system | 18 weeks | 29.08.2016 | 406.4 ± 66.8 | 1.14 ± 0.06 | 7.5 → 12 |
| Study 4 F12 | 59° 57′ 49.8′′N | 5° 49′ 23.8′′E | 28.04.2016 | Netpen | 18 weeks | 30.08.2016 | 543.6 ± 246.1 | 1.10 ± 0.20 | 8.5 → 15 |
| Study 5 F13 | n/a | n/a | n/a | n/a | n/a | 02.07.2018 | n/a | n/a | n/a |
| Study 5 F14 | n/a | n/a | n/a | n/a | n/a | 16.08.2018 | n/a | n/a | n/a |
| Study 5 F15 | n/a | n/a | n/a | n/a | n/a | 16.08.2018 | n/a | n/a | n/a |

FT = flow through. *salinity 25ppm. ** arrows indicate that water temperature was not fixed but followed seasonal variation in the duration of the experiment, which length is indicated in the column “diet duration”.

**Supplementary Table S2.** Overview of microbiome samples and sequence output

|  | | **Number of samples sequenced per sample category*** | | | | **Mean number of ASVs (amplicon sequence variants) per sample category following filtration** | | | |
| --- | --- | --- | --- | --- | --- | --- | --- | --- | --- |
| **Study and feed** | **Seq. run (year)** | **Feed** | **PID** | **MID** | **DID** | **Feed** | **PID** | **MID** | **DID** |
| Study 1 F1 | 2 (2019), 4 (2019) | 3 | 3 (5) | 2 (4) | 3 (13) | 154252 | 8220 | 5787 | 55737 |
| Study 1 F2 | 2 (2019), 4 (2019) | 3 | 3 (14) | 3 (8) | 3 (15) | 88995 | 1537 | 6253 | 47279 |
| Study 2 F3 | 3 (2019) | 3 |  |  | 5 | 48031 |  |  | 112279 |
| Study 2 F4 | 3 (2019) | 3 |  |  | 5 | 54221 |  |  | 94586 |
| Study 2 F5 | 3 (2019) | 3 |  |  | 5 | 33991 |  |  | 39323 |
| Study 3 F6 | 1 (2018) | 1** |  |  | 3 (9) | 212500 |  |  | 159055 |
| Study 3 F7 | 1 (2018) | 1** |  |  | 3 (7) | 183403 |  |  | 119669 |
| Study 3 F8 | 1 (2018) | 1** |  |  | 3 (6) | 182514 |  |  | 130840 |
| Study 3 F9 | 1 (2018) | 1** |  |  | 3 (7) | 150493 |  |  | 78976 |
| Study 3 F10 | 1 (2018) | 1** |  |  | 3 (9) | 168933 |  |  | 134769 |
| Study 4 F11 | 2 (2019) | 3 |  |  | 5 | 31034 |  |  | 24686 |
| Study 4 F12 | 2 (2019) | 3 |  |  | 5 | 22786 |  |  | 52893 |
| Study 5 F13 | 2 (2019) | 3 |  |  | nd | 44088 |  |  |  |
| Study 5 F14 | 2 (2019) | 3 |  |  | nd | 30893 |  |  |  |
| Study 5 F15 | 2 (2019) | 3 |  |  | nd | 33508 |  |  |  |

| ^*^The numbers indicate the final merged samples, in total 92, and numbers in parenthesis indicate the total numbers of unique samples sequenced. ^**^Based on merged sequence data from replica DNA extraction, PCR, and sequences from one original feed sample. nd, not determined/not sequenced due to low amounts of DNA and PCR bands detected. |
| --- |
|  |

**Sample collection of feed and intestinal digesta**

Atlantic salmon were netted and immediately killed by either a lethal dose of tricaine (Finquel, Scan Aqua) or benzocaine (Benzoak Vet, ACD Pharmaceuticals), dependent on the site location, before instantly sampled on site. The abdominal cavity was opened, and the whole intestine was aseptically removed from the abdominal cavity. In study 1, the intestine was separated into the proximal, mid, and distal intestinal compartments as described by others [2]. The intestinal content (digesta) was collected separately from all three sections. In study 2-4, only the distal intestinal region was sampled. A single-use sterile scalpel blade was used to make an incision to open the intestinal compartments. Digesta collection was performed using a new scalpel blade to collect bulk faeces, excluding the intestinal mucus layer. Dry feed pellets were secured from the different feeding systems. Samples were stored in RNAlater™ (Invitrogen) and, after 24 hours, maintained at -80 °C until DNA extraction.

**Nucleic acid extraction**

DNA was extracted from ~100 mg feed pellets or intestinal digesta. The protocol was performed using the PowerLyzer® PowerSoil® DNA Isolation Kit (MoBio) according to the manufacturer’s specification with the following amendments: samples after adding Solution C1 were heated at 70 °C for 10 min. Samples were homogenized with the mechanical bead beater device Precellys®24 (Bertin Technologies) for 2 x 20 s at 5000 rpm. The DNA was re-suspended in 50 μl of DNase/RNase free molecular water, and concentration determined using a Thermo Scientific Nanodrop 2000c.

**Microbiome analysis**

Overview of all feed and digesta samples used for the microbiome analysis is shown in Table S2. The extracted DNA samples were analyzed by 16S rRNA gene amplicon sequencing (2×150 bp) of the variable region 4, as described elsewhere [3] and used in the Earth Microbiome Project [4]. The current primers [5-7] have been modified from the original 515F–806R primer pair, with barcodes now on the forward primer and degeneracy added to both the forward and reverse primers to remove known biases. In total, four sequencing runs were done on a MiSeq (Illumina) at Nofima using pooled polymerase chain reaction (PCR) samples, which were based on triplicate PCRs per DNA sample using sample-specific barcoded forward primers. Agarose gel separation was used to confirm amplification of the target region and negative amplification of negative controls. PhiX Control v3 was included in the sequencing runs and accounted for 10% of the reads. The MiSeq Control Software (MCS) version used was RTA 1.18.54.

Data processing of the sequencing reads was performed using the pipelines in QIIME2 (version 2020.2) [8]. Briefly, the data from each sequencing run was separately demultiplexed and paired ends joined, quality filtered and denoised using dada2, before the four data sets were merged, and taxonomy was achieved using classify-sklearn with the SILVA 132 database (132-99-515-806). Each unique taxonomic feature represents an amplicon sequence variant (ASV), and ASVs were filtered out when assigned to Chloroplast or Mitochondria (accounted for more than half of the feature counts), were less frequent than 100 or observed in less than three samples. ASV data from digesta samples collected from individual fish of the same tank origin (study 1 and 3) were merged to obtain more balanced number of DID samples per feed across the studies. Samples with less than 1000 ASVs were excluded. This resulted in an ASV table with 92 samples containing 649 unique ASVs from a total of 6.4 million ASV counts. The ASV table was used for alpha diversity rarefaction analysis using an equal number of ASVs across samples (i.e. 10,000 sequences per sample). Taxonomic summary table at genus level was used for further analysis with the 40 genera or last known taxa with a mean relative abundance >0.1% across the samples. The genera *Prevotella* 7 and *Prevotella* 9 were merged into one *Prevotella* 7&9, and *Harryflintia* was renamed to *Ruminococcaceae* since its representative ASV was taxonomically determined with only 90% taxonomic confidence in the SILVA database. Usually one ASV dominated per genera, which was also the case for *Photobacterium*, with >99% confidence. One exception was *Lactobacillus*, that was dominated by several ASVs (Fig. S5). Similar overlap was observed between feed and DID microbiomes both at ASV and genus level, also when taking into account the different ASVs for *Lactobacillus*, and therefore the data was presented at genus level. The frequency of dominating genera in the feed or DIDs was estimated as how often the genera were detected as dominant (>1%) in the different feed types (n=15) or in the different DID types (n=12).

Metagenomic sequencing of DNA from feed F5 and respective DID (study 2) was performed on Hiseq4000 (Illumina) applying pair-end 150 bp. The DNA was quantified by Qubit (Invitrogen) and prepped using KAPA HyperPrep (Roche). The sequencing service was provided by the Norwegian Sequencing Centre (www.sequencing.uio.no), a national technology platform hosted by the University of Oslo and supported by the “Functional Genomics” and “Infrastructure” programs of the Research Council of Norway and the Southeastern Regional Health Authorities. Sequences were quality checked with FastQC v0.11.9 [9], which identified a varying degree of adapter contamination in the samples. Adapter contaminations were removed using bbduk (included in bbmap 38.87, https://jgi.doe.gov/data-and-tools/bbtools/) with the included universal adapter and primer collection for Illumina kits using the parameters: “ktrim=r k=23 mink=11 hdist=1 tpe tbo”. Quality checked and trimmed reads were annotated using the taxonomic classification tool Kaiju v1.7.4 [10] against the nr_euk database (NCBI non-redundant sequences, which includes Archea, Bacteria, Virus and Eucaryotic divisions). All samples were explored using MEGAN 6 community edition [11]. Only classified reads from Kaiju were imported, then the total number of reads was set manually for each sample after input. Samples were compared using normalized counts. The Core-microbiome was computed (100% overlap between samples, min 1% abundance) for each sample. Exported core microbiomes were converted to Graphlan input using the mergetables utility script from Metaphlan.

**Statistical analysis**

Principal components analyses (PCA) and hierarchical cluster analyses were used as pattern recognition algorithms to examine feed, and intestinal digesta (PID, MID, and DID) data sets using the relative abundance values of the 40 most abundant taxonomic groups for cluster information using ClustVis [12]. The relative abundance for the 40 most abundant taxa of feed and intestinal profiles were compared by calculating Pearson *r* correlation coefficient using JMP® Pro 13.1.0. (SAS Institute Inc. software). Values *p* < 0.05 were considered significant.

**Accession number**

Microbiota data were exported as individual fastq files and has been deposited in the Sequence Read Archive (SRA) of the National Center for Biotechnology Information (NCBI) under the accession number PRJNA791377.

**Supplementary Figures**

**Fig. S1.** Alpha diversity shown as number of observed ASVs within the feed and DID samples. Mean observed ASVs within the feeds (*n*=15) and DIDs (*n*=12) were 140±38 and 125±93, respectively.


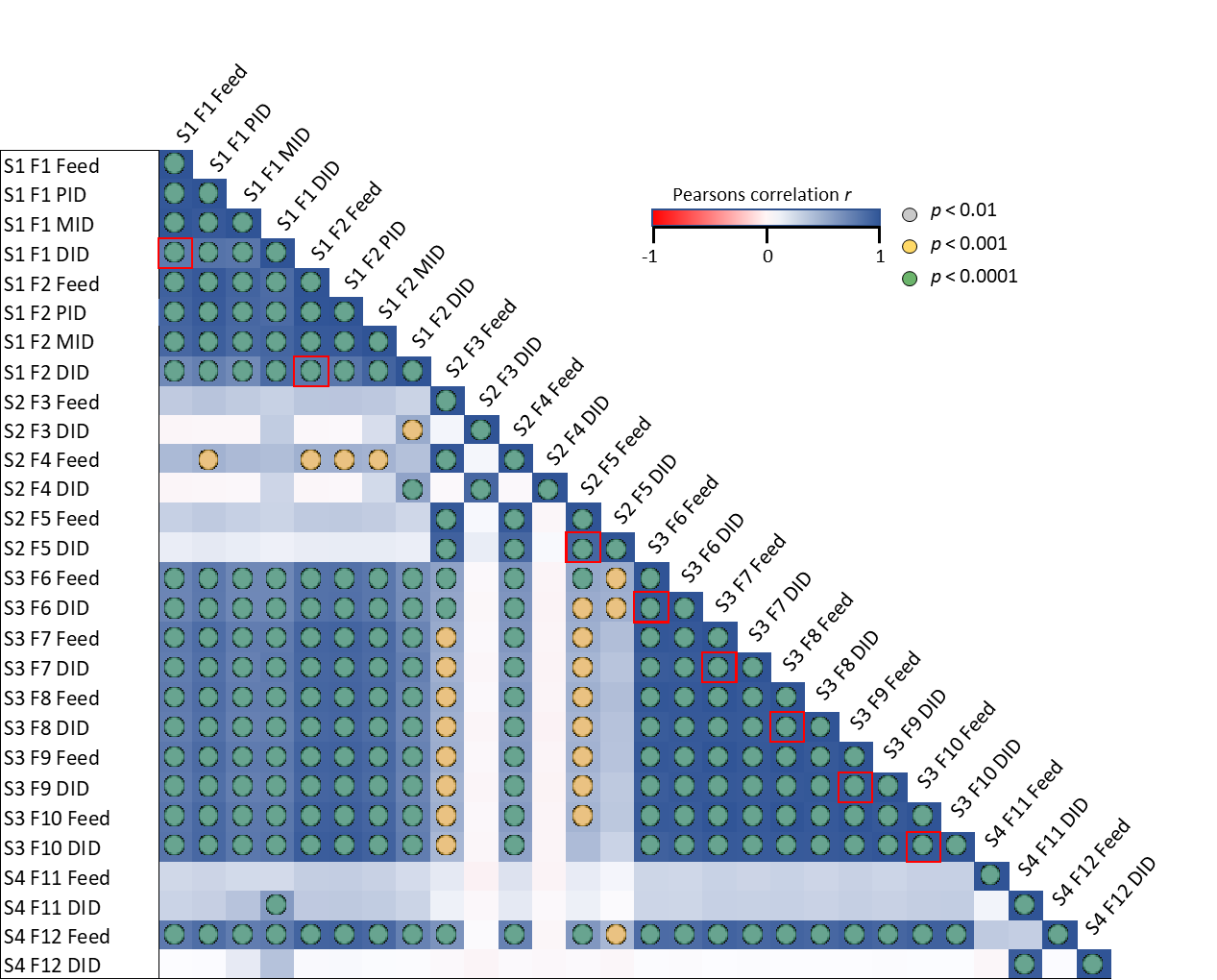


**Fig. S2.** Correlation analysis of the microbial composition between the Atlantic salmon feeds and proximal intestinal digesta (PID), mid intestinal digesta (MID), and distal intestinal digesta (DID) in the representative case studies (S1-S4) and feeds (F1-F12). The color indicates the value and direction of the Pearson correlation coefficient. Only significant (*p*<0.05) correlations are shown. The red boxes highlight a significant correlation of feed with their respective distal intestinal digesta (DID). In study S1, higher correlation (Pearson's r) values were observed between the feed and PID and MID, compared to feed and DID microbiome profiles. Findings from study S1-S4 suggest a model wherein microbial profiles are to a different degree facilitated by bacterial DNA present in the feed itself. The level of Pearson correlation significance is indicated by colored circles: grey circle *p* < 0.01, yellow circle *p* < 0.001, green circle *p* < 0.0001.

>7894cb48136ee2d0893a37eca6c528ae

AGGGTGCGAGCGTTAATCGGAATTACTGGGCGTAAAGCGCATGCAGGCGGTCTGTTAAGCAAGATGTGAAAGCCCGGGGCTCAACCTCGGAACAGCATTTTGAACTGGCAGACTAGAGTCTTGTAGAGGGGGGTAGAATTTCAGGTGTAGCGGTGAAATGCGTAGAGATCTGAAGGAATACCGGTGGCGAAGGCGGCCCCCTGGACAAAGACTGACGCTCAGATGCGAAAGCGTGGGGAGCAA

**Fig. S3.** Fasta sequence of the dominating ASV assigned to *Photobacterium*.


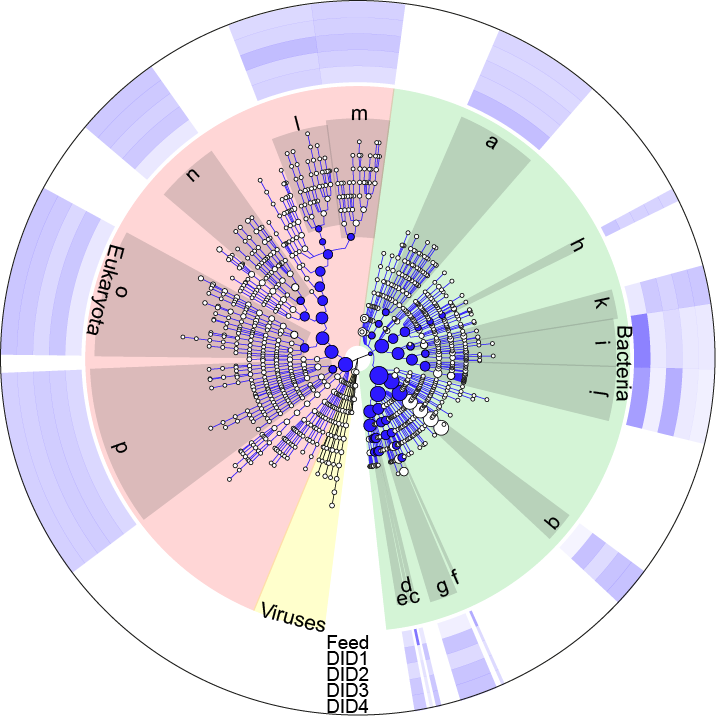


**Fig. S4.** The metagenome produced by GraPhlAn software with metadata from MetaPhlAn combining the feed and DID from the cohort study 2 and feed F5. The grey gradient indicates at what taxonomic level sequences have been assigned with the different taxa marked by lowercase letters: (a) Bacteroidetes, (b) Rhizobiales, (c) *Escherichia coli,* (d) *Klebsiella pneumoniae*, (e) Unclassified *Salmonella*, (f) *Acinetobacter baumannii*, (g) *Pseudomonas*, (h) *Streptomyces*, (i) Bacillales, (j) Lactobacillales, (k) Clostridia, (l) Dothideomycetes, (m) Sordariomyceta, (n) Agaricomycotina, (o) Fungi *Incertae sedis*, (p) Sar. The taxonomic cladograms report all nodes from taxonomic classification of samples in Kaiju with circle size proportional to the log of average abundance. Core microbiome between feed and DID is color coded blue and represents relative enrichment of the most abundant taxa (100% sequence overlap between samples present ≥1% of the total abundance).


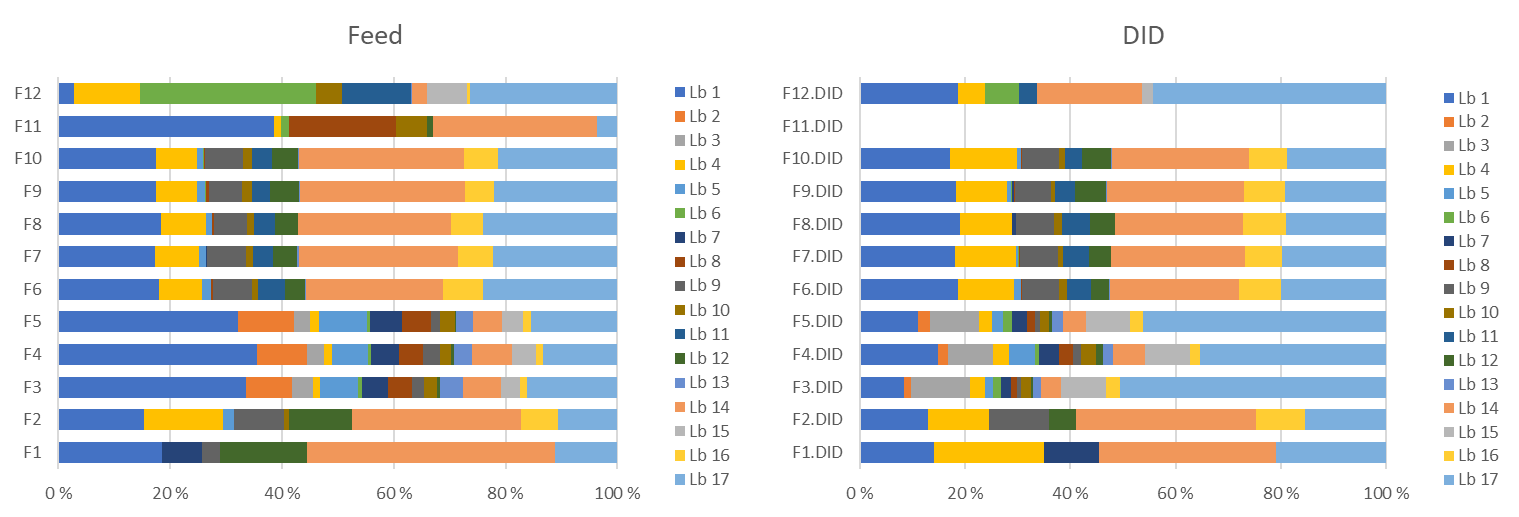


**Fig. S5.** Relative distribution of different ASVs assigned to *Lactobacillus* (Lb1-Lb17) within 12 different feeds (F1-F12) and within the respective DIDs (F1.DID-F12.DID) compared to total *Lactobacillus* ASVs.

**Supplementary Information References**

1. Karlsen C, Ytteborg E, Timmerhaus G, Høst V, Handeland S, Jørgensen SM, Krasnov A: **Atlantic salmon skin barrier functions gradually enhance after seawater transfer**. *Scientific Reports* 2018, **8**(1):9510.

2. Gajardo K, Rodiles A, Kortner TM, Krogdahl Å, Bakke AM, Merrifield DL, Sørum H: **A high-resolution map of the gut microbiota in Atlantic salmon (*Salmo salar*): A basis for comparative gut microbial research**. *Scientific Reports* 2016, **6**:30893.

3. Caporaso JG, Lauber CL, Walters WA, Berg-Lyons D, Huntley J, Fierer N, Owens SM, Betley J, Fraser L, Bauer M *et al*: **Ultra-high-throughput microbial community analysis on the Illumina HiSeq and MiSeq platforms**. *Isme j* 2012, **6**(8):1621-1624.

4. Gilbert JA, Jansson JK, Knight R: **The Earth Microbiome project: successes and aspirations**. *BMC Biol* 2014, **12**:69.

5. Walters W, Hyde ER, Berg-Lyons D, Ackermann G, Humphrey G, Parada A, Gilbert JA, Jansson JK, Caporaso JG, Fuhrman JA *et al*: **Improved bacterial 16S rRNA gene (V4 and V4-5) and fungal internal transcribed spacer marker gene primers for microbial community surveys**. *mSystems* 2016, **1**(1).

6. Parada AE, Needham DM, Fuhrman JA: **Every base matters: assessing small subunit rRNA primers for marine microbiomes with mock communities, time series and global field samples**. *Environ Microbiol* 2016, **18**(5):1403-1414.

7. Apprill A, McNally S, Parsons R, Weber L: **Minor revision to V4 region SSU rRNA 806R gene primer greatly increases detection of SAR11 bacterioplankton**. *Aquatic Microbial Ecology* 2015, **75**.

8. Bolyen E, Rideout JR, Dillon MR, Bokulich NA, Abnet CC, Al-Ghalith GA, Alexander H, Alm EJ, Arumugam M, Asnicar F *et al*: **Reproducible, interactive, scalable and extensible microbiome data science using QIIME 2**. *Nature Biotechnology* 2019, **37**(8):852-857.

9. Andrews S: **FastQC: A Quality Control Tool for High Throughput Sequence Data [Online]. Available online at:** [**http://www.bioinformatics.babraham.ac.uk/projects/fastqc/**](http://www.bioinformatics.babraham.ac.uk/projects/fastqc/). 2010.

10. Menzel P, Ng KL, Krogh A: **Fast and sensitive taxonomic classification for metagenomics with Kaiju**. *Nature Communications* 2016, **7**(1):11257.

11. Huson DH, Beier S, Flade I, Górska A, El-Hadidi M, Mitra S, Ruscheweyh H-J, Tappu R: **MEGAN Community Edition - Interactive exploration and analysis of large-scale microbiome sequencing data**. *PLOS Computational Biology* 2016, **12**(6):e1004957.

12. Metsalu T, Vilo J: **ClustVis: a web tool for visualizing clustering of multivariate data using Principal Component Analysis and heatmap**. *Nucleic Acids Research* 2015, **43**(Web Server issue):W566-W570.
